# Salivary antibodies induced by the seven-valent PncOMPC conjugate vaccine in the Finnish Otitis Media Vaccine Trial

**DOI:** 10.1186/1471-2334-5-41

**Published:** 2005-05-27

**Authors:** Anu Nurkka, Mika Lahdenkari, Arto AI Palmu, Helena Käyhty, the FinOM Study Group

**Affiliations:** 1 Department of Vaccines, National Public Health Institute (KTL), Helsinki, Finland

## Abstract

**Background:**

Mucosal antibodies have been suggested to have a role in defence against pneumococcal infections. We investigated here the ability of a seven-valent pneumococcal conjugate vaccine, PncOMPC, to induce mucosal immune response.

**Methods:**

Healthy Finnish children (n = 111), a subcohort of the Finnish Otitis Media Vaccine Trial, were recruited and 56 of them were immunised with the PncOMPC at the age of 2, 4, and 6 months. At 12 months of age, 49 of them received the PncOMPC and 7 were vaccinated with the pneumococcal polysaccharide vaccine (PncPS) as a booster. The control group of 55 children received a hepatitis B vaccine at the same ages. Salivary anti-Pnc IgG, IgA, IgA1, and IgA2 antibodies to serotypes 6B, 14, 19F, and 23F were measured in both groups at the age of 7 and 13 months.

**Results:**

Salivary anti-Pnc IgG and IgA were detected more often in the PncOMPC than in the control group. However, the difference between groups was significant only for 19F and 23F IgA concentrations at the age of 7 months. At the age of 13 months, antibody concentrations did not differ between PncOMPC and control groups. The rises in IgA concentrations between 7 and 13 months of age were mainly of subclass IgA1. Further, there is a clear trend that PncPS booster induces higher salivary anti-Pnc PS antibody concentrations than the PncOMPC.

**Conclusion:**

We found that PncOMPC can induce a mucosal IgA response. However, the actual impact of mucosal antibodies in protection against pneumococcal infections is not clear.

## Background

*Streptococcus pneumoniae *(Pnc) frequently colonises mucosal epithelium at nasopharynx without causing any symptoms [1]. The carriage rate varies depending on the age, being highest in children under two years of age [2]. Further, the prevalence of pneumococcal carriage is higher in developing than developed countries [3]. Although the pneumococcal carriage is often harmless, it may lead to a local disease, e.g. to acute otitis media (AOM), sinusitis or to an invasive disease like pneumonia, meningitis or sepsis [3].

The main mechanism of defence against pneumococcus are anticapsular antibodies, which help in the phagocytosis or which can counteract acquisition of pneumococcus probably by preventing adhesion to the mucosal surface [4]. In serum the predominant immunoglobulin class is IgG. The salivary IgG is mainly derived from serum by leakage across capillaries and entering saliva through gingival crevices. However, some local production of IgG may take place [5,6]. At mucosal membranes IgA is the main immunoglobulin class and it is found most often in the secretory form (sIgA). The role of serum IgG in the defence against pneumococcus is obvious; IgG can activate complement efficiently and further lead to phagocytosis of bacteria. The function of mucosal antibodies in humans is less clear. However, there are several pieces of evidence, which suggest that they do have a role in the defence. The presence of pneumococcus in nasopharynx induces salivary antibodies against pneumococcal proteins and polysaccharides already in infants [7,8], and pneumococcal conjugate vaccines evoke mucosal immune response [5,9-15]. In addition to invasive disease and pneumonia, pneumococcal conjugate vaccines prevent also local infections like AOM and carriage [16-22]. Further, in an animal model mucosal antibodies prevented the acquisition of pneumococcus [23].

At the moment there is only one licensed pneumococcal conjugate vaccine, PncCRM. The vaccine contains seven pneumococcal capsular polysaccharides (PS) conjugated to a non-toxic variant form of diphtheria toxin (CRM_197_). The Kaiser Permanente Efficacy Trial in the USA showed that PncCRM is highly protective, 97.4% (95% CI 88.7 to 99.9%), against invasive pneumococcal disease caused by vaccine serotypes [16]. Among American Indian children, which are a high risk population for pneumococcal infection, the intention-to-treat total primary efficacy of PncCRM against invasive disease was 82.6% (95% CI 21.4 to 96.1%) [24]. The efficacy of a 9-valent PncCRM in HIV-infected and uninfected children has been studied in Soweto, South-Africa [25]. The vaccine prevented there 83% (95% CI 39–97%) of invasive pneumococcal infections due to vaccine serotypes in HIV-uninfected children. The respective number was 65% (95% CI 24–86%) in HIV-infected children.

In the Finnish Otitis Media (FinOM) Vaccine Trial, two pneumococcal conjugate vaccines, PncCRM and PncOMPC, were investigated in parallel regarding the efficacy against AOM using hepatitis B vaccine (HBV) as a control for both arms. The efficacy of the PncCRM vaccine was 57% (95% CI 44 to 67%) [19] and of the PncOMPC vaccine 56% (95% CI 44 to 66%) [22] against AOM caused by the vaccine serotypes. In the setting of the FinOM Vaccine Trial we have had a possibility to evaluate both the serum [19,22] and mucosal immunity [10] as surrogates of vaccine efficacy [26].

The aim of this study was to measure the mucosal antibody responses after vaccination with 3 or 4 doses of either the PncOMPC or the control vaccine. Measurement of anti-Pnc antibodies against serotypes 6B, 14, 19F and 23F were selected because these types were most commonly associated with AOM in the FinOM studies [10,19,27]. We compared the prevalence and concentrations of salivary antibodies at 7 and 13 months of age in the PncOMPC and control group. We also explored the effect of pneumococcal polysaccharide vaccine (PncPS) as a booster. The data on salivary antibody in the PncCRM group has been reported separately [10].

## Methods

### Vaccines, vaccinees and vaccinations

PncOMPC (Merck & Co., Inc., West Point, PA) is a heptavalent pneumococcal conjugate vaccine containing 5 μg of capsular PS of the serotype 6B, 3 μg of type 23F, 2 μg of types 18C and 19F, 1.5 μg of type 9V, and 1 μg of types 4 and 14 each conjugated individually to an outer membrane protein complex (OMPC) of *Neisseria meningitidis *serogroup B. Aluminium was used as an adjuvant and thiomersal as a preservative. Pneumovax^® ^(Merck & Co., Inc.) is a 23-valent pneumococcal PS vaccine (PncPS), containing 25 μg of each capsular PS. HBV (Recombivax ™ Merck & Co., Inc.) was used as a control vaccine. One dose of HBV vaccine contains 5 μg of hepatitis B surface antigen.

The design of the FinOM Vaccine Trial has been described previously [19,22]. In this study we had a subcohort of 111 infants. 56 children were randomised to receive the PncOMPC vaccine at the age of 2, 4, and 6 months. At 12 months of age, 49 of them were vaccinated with the PncOMPC and 7 with the PncPS as a booster vaccine. The control group of 55 children received HBV at the same ages.

Additionally, the children received a combined diphtheria-tetanus-whole cell pertussis and *Haemophilus influenzae *type b conjugate vaccine (Tetramune^®^, Wyeth Pharmaceuticals) at 2, 4, 6, and 24 months, inactivated polio vaccine (Imovax^®^, Aventis Pasteur) at 7, 12, and 24 months and measles-mumps-rubella (MMR^®^II, Merck & Co., Inc.) at 18 months of age.

### Sampling

Unstimulated saliva samples were taken at the age of 7 (n = 111) and 13 (n = 107) months. Mothers were advised not to breast feed infants during one hour before saliva sampling. Up to 2 ml of saliva was collected with a gentle aspiration using electronic suction device and samples were immediately frozen at -70°C. Before analysis the samples were centrifuged for 10 minutes with 15 000 rpm and the supernatant was used for analyses of anti-Pnc PS antibodies. The saliva samples were thawed only once. The volume of the sample was not always sufficient for all analyses.

### Measurement of antibodies

#### Enzyme immunoassay (EIA) for saliva samples

IgA, IgA1, IgA2, and IgG antibodies to serotypes 6B, 14, 19F and 23F were measured by EIA as described previously [5,10,11,14].

Before calculation of concentrations, optical density (OD) readings from the PBS wells were subtracted from the antigen plates. For IgA and IgG results OD values of ≥ 0.05 (≥ 2 standard deviations of the blank) were regarded as positive. The concentrations of IgA and IgG were calculated in nanograms per millilitre (ng/ml) of saliva by using the 89-SF serum as a reference. The detection limit was 5 ng/ml for both IgA and IgG assays and for all serotypes. Samples with undetectable IgA and IgG were assigned a value 1.7 ng/ml; half a log less than the detection limit. The IgA1 and IgA2 results are presented as EIA units/ml calculated using 89-SF as a reference with a given calibration factor [28]. The detection limit of IgA1 and IgA2 assays for all the serotypes was 1.3 units/ml; and samples with undetectable IgA1 and IgA2 concentrations were given a value 0.65 units/ml.

### Statistical methods

Results are given as geometric mean antibody concentrations (GMC) with 95% confidence intervals (CI). Differences in antibody concentrations in saliva samples between different ages were analysed with Wilcoxon Signed Ranks Test. Kruskal-Wallis test was used to compare differences between PncOPMC and HBV groups. Proportions of children having anti-Pnc antibodies in saliva were compared with Yates-corrected chi square test (χ^2^-test) or with Fisher's two-tailed exact test. Differences were considered significant when p-value was <0.05. The sample size was not specifically determined for this study.

## Results

### Anti-pneumococcal IgG in saliva

Anti-Pnc PS IgG was detected at the age of both 7 and 13 months very rarely, and there were no differences either in the proportion of positive samples or concentration between the PncOMPC and control groups except for serotype 14 at 13 months; there were more anti-Pnc PS IgG positive samples in the PncOMPC than in the control group (Table 1).

**Table 1:** Salivary anti-Pnc PS IgG antibodies in the PncOMPC, PncOMPC+PncPS booster and control groups; number and the proportion of positive samples at the age of 7 and 13 months.

Pnc serotype	Age	IgG			
		PncOMPC group		Control group	
		
		N, samples	Number positive (%)	N, samples	Number positive (%)
6B	7 mo	56	1 (2)	55	0
	13 mo (PncOMPC booster)	44	3 (7)	55	0
	13 mo (PncPS booster)	6	1 (17)	NA	

14	7 mo	56	2 (4)	55	2 (4)
	13 mo (PncOMPC booster)	44	6 (14)^a^	55	1 (2)
	13 mo (PncPS booster)	6	3 (50)	NA	

19F	7 mo	56	2 (4)	54	5 (9)
	13 mo (PncOMPC booster)	44	6 (14)	55	2 (4)
	13 mo (PncPS booster)	5	3 (60)	NA	

23F	7 mo	56	1 (2)	54	1 (2)
	13 mo (PncOMPC booster)	44	0	55	1 (2)
	13 mo (PncPS booster)	6	0	NA	

^a ^statistical difference in the number of positive samples between PncOMPC and control groups, p = 0.04.

A small group of 7 children received PncPS vaccine as a booster instead of PncOMPC at the age of 12 months. Further, the volume of saliva was not sufficient for all the EIA analyses. However, there was a clear trend that the PncPS boosting induced more often IgG to the saliva than the PncOMPC boosting (Table 1).

### Anti-pneumococcal IgA in saliva

**Figure 1:**
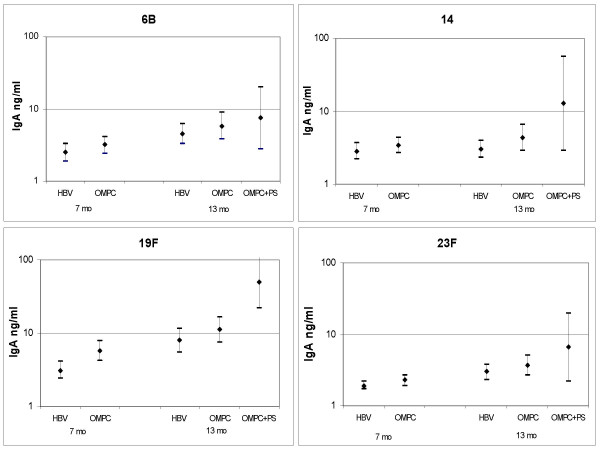
Salivary anti-Pnc PS IgA concentrations (ng/ml) with 95% confidence intervals in the PncOMPC, PncOMPC+PncPS booster and control groups at the age of 7 and 13 months.

At the age of 7 months, anti-Pnc PS IgA was detected more often and the concentrations were higher in the PncOMPC than in the control group for all four serotypes (Table 2, Fig 1). However, the difference was statistically significant only for serotypes 19F (both the number of positive samples and concentration) and 23F (concentration). The geometric mean concentrations (GMCs) varied between 2.3 and 5.7 ng/ml, and 1.9 and 3.1 ng/ml in the PncOMPC and control groups, respectively (Fig 1).

**Table 2:** Salivary anti-Pnc PS IgA antibodies in the PncOMPC, PncOMPC+PncPS booster and control groups; number and the percentage of positive samples at the age of 7 and 13 months.

Pnc serotype	Age	IgA			
		PncOMPC group		Control group	
		
		N, samples	Number positive (%)	N, samples	Number positive (%)
6B	7 mo	56	17 (30)	55	9 (16)
	13 mo (PncOMPC booster)	45	23 (51)	55	26 (47)
	13 mo (PncPS booster)	7	5 (71)	NA	

14	7 mo	56	23 (41)	55	14 (25)
	13 mo (PncOMPC booster)	45	19 (42)	55	16 (29)
	13 mo (PncPS booster)	7	5 (71)	NA	

19F	7 mo	56	32 (57)^a^	55	17 (31)
	13 mo (PncOMPC booster)	45	35 (78)	55	35 (64)
	13 mo (PncPS booster)	7	7 (100)	NA	

23F	7 mo	56	11 (20)	55	3 (5)
	13 mo (PncOMPC booster)	45	18 (40)	55	17 (31)
	13 mo (PncPS booster)	7	5 (71)	NA	

^a ^statistical difference in the number of positive samples between PncOMPC and control groups, p = 0.01.

At the age of 13 months, there were no statistical differences between the PncOMPC and the control group either in the proportion of positive samples or in the anti-Pnc PS IgA concentrations (Table 2, Fig 1). After the booster, the GMCs ranged between 3.7 and 11.2 ng/ml, and 3.0 and 8.0 ng/ml in the PncOMPC and control group, respectively. However, there was a statistical difference between 7 and 13 months of age in the anti-Pnc PS IgA concentrations for serotype 23F in the PncCRM group and for serotypes 6B, 19F and 23F in the HBV group.

PncPS booster induced higher anti-Pnc PS IgA concentrations than the PncOMPC (Table 2, Fig 1). However, the small number of samples in the PncPS group did not allow any statistical analyses.

### IgA1 and IgA2 subclasses

**Figure 2:**
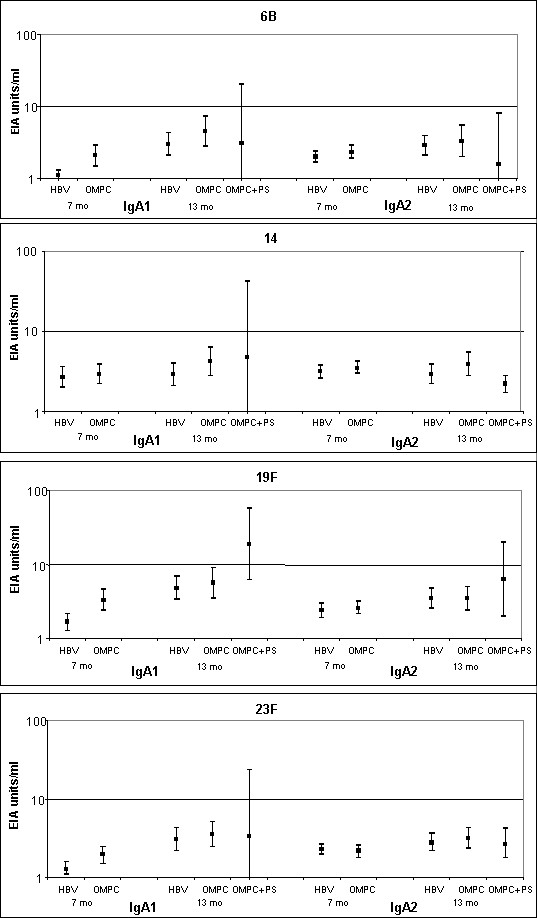
Salivary anti-Pnc PS IgA1 and IgA2 concentrations (EIA units/ml) with 95% confidence intervals in the PncOMPC, PncOMPC+PncPS booster and control groups at the age of 7 and 13 months.

At the age of 7 months, IgA1 concentrations were mirroring the total anti-Pnc serotype specific IgA (Fig 1) and were significantly higher in the PncOMPC than in the control group for serotypes 6B, 19F, and 23F. However, at the age of 13 months, there were no statistical differences between the groups. The IgA2 concentrations did not differ between the groups either at the age of 7 or 13 months. Thus, rises in the antibody concentrations were seen only for IgA1 (Fig 2).

## Discussion

Salivary anti-Pnc polysaccharide antibodies, both IgG and IgA, were detected only slightly more often in the PncOMPC than in the control group. However, the proportion of positive samples and the antibody concentrations rose between 7 and 13 months of age in both groups and the differences between the vaccine groups diminished. This suggests that children in both groups had pneumococcal contacts which induced development of mucosal antibodies. This is in accordance with the findings that pneumococcal carriage induces salivary antibodies in children [7], also regardless of the previous pneumococcal vaccination status [29].

In addition to IgA, we measured also IgA subclass specific antibody development. IgA1 is the predominant subclass both in serum and saliva, IgA1:IgA2 ratios being roughly 9:1 in serum and 6:4 in saliva [30]. To improve their virulence, some bacteria including pneumococcus have developed proteases, which degrade IgA1 antibodies [31]. Recently, IgA1-proteases have been found to be important in the adherence of pneumococcus [32]. Due to methodological reasons the IgA1 and IgA2 concentrations detected in this study can not be compared as the data are given in EIA units that are not comparable. However, we found that the changes and the difference in the IgA concentrations were mainly due to changes in the IgA1 concentrations. This is in accordance with our previous findings [11,33]. This suggests that both IgA1 and IgA2 are present, but the vaccine induces IgA1, which, unfortunately, is susceptible to IgA proteases.

We have also reported the mucosal anti-Pnc PS antibodies in the PncCRM arm of the FinOM Vaccine Trial [10]. Though the PncCRM induced more often and higher concentrations of salivary anti-Pnc PS antibodies than the PncOMPC vaccine (all the samples were analysed together at the same time), the basic message from the two studies is the same. The data of this study and of the PncCRM arm [10] suggest that parenteral immunization with pneumococcal conjugate vaccines induce local IgA1 antibody formation, but the antibodies persist only for a short period. The concentrations of anti-Pnc PS IgA at 13 months and in the PncCRM arm also at 4 to 5 years of age were very similar in the pneumococcal vaccine and in the control vaccine groups indicating that no remarkable natural boosting of the vaccine-induced antibodies occurred [10]. IgG anti-Pnc PS antibodies were detected only rarely. The highest prevalence was found in the small group of children whose 4^th ^pneumococcal vaccine dose at 12 months was PncPS vaccine. This is in accordance with the serum antibody concentrations that were significantly higher in the PncPS-boosted than in the PncOMPC-boosted children for all other serotypes except 23F [34].

Pneumococcal PS vaccine has been suggested to be used as a booster after a primary series with a pneumococcal conjugate vaccine. It is cheaper than the conjugate vaccine available and induces higher serum antibody concentrations than a conjugate booster [14,22,34]. However, a conjugate vaccine as a booster can be favourable for the persistence and quality of antibodies, because it stimulates the formation of high-affinity clones of memory B-cells [35]. There is also a possibility that PS booster may cause depletion of memory cells [36]. Further, no significant differences in the efficacy against AOM and recurrent AOM have been found between PS and conjugate booster [22,37]. We found in this study, that there is a clear trend that the PS vaccine as a booster induces mucosal anti-pneumococcal antibodies more often and in higher concentration than conjugate booster. The same effect of a polysaccharide booster in salivary antibody concentrations have been seen also previously [9,14]. However, there may be differences in the quality of salivary antibodies induced by the PS or conjugate vaccines.

Pneumococcal conjugate vaccines have been found to induce both humoral and mucosal immunity. Further, the vaccines have been found effective against invasive and local pneumococcal infections. However, the antibody concentrations or quality of antibodies needed for protection against disease and carriage are not known, even if there has been a lot of discussion about the protective levels [38,39]. Recently, a consensus about the estimate of serological surrogate of protection against invasive pneumococcal infections has been achieved. It is suggested that the 0.35 μg/ml concentration of anti-Pnc PS IgG in serum would predict the protection against pneumococcal infection at the population level . However, the value is a rough estimate and further, the function of antibodies, both opsonophagocytic activity and avidity, and immunological memory are also essential issues in serological immunity. In addition to the concentration and function of anti-pneumococcal antibodies in serum, the importance and function of mucosal anti-pneumococcal antibodies should be clarified. In the FinOM Vaccine Trial the PncCRM induced more salivary antibodies than the PncOMPC, but the efficacy of these vaccines against AOM was similar. This raises a question, which is the actual mechanism of protection? Anyway, we know that pneumococcal carriage, infections and conjugate vaccines all induce mucosal antibodies. But we do not know how important mucosal antibodies really are in the defence against pneumococcal infections compared with the antibodies in serum, and if mucosal immunisation would provide better local immune response.

## Conclusion

We found that PncOMPC can induce a salivary IgA response. However, the actual impact of mucosal antibodies in protection against pneumococcal infections is not clear.

## Competing interests

A Palmu has had travel paid for by Wyeth-Lederle and GlaxoSmithKline as an invited speaker at symposia and received an honorarium from Wyeth-Lederle. H Käyhty has provided consultancies on advisory boards for Aventis Pasteur, GlaxoSmithKline and ID Biomedical Corporation; has had travel paid for by Aventis Pasteur, GlaxoSmithKline, Spectrum Medical Sciences and Wyeth Lederle Vaccines as an invited speaker or expert at symposia; and has received honoraria from Aventis Pasteur, GlaxoSmithKline, and Wyeth Lederle Vaccines. The other authors declare no competing interests.

## Authors' contributions

AN conducted the laboratory analyses and analysed the immunogenicity data. ML was responsible for the statistical analyses. AP was the study coordinator responsible for enrolment, clinical evaluation and saliva sampling of the study subjects and participated in the planning of study design together with the FinOM Study Group. HK supervised the immunogenicity analyses and participated in the planning of study design together with the FinOM Study Group. All authors contributed to the writing of the manuscript and approved the final version.

## Pre-publication history

The pre-publication history for this paper can be accessed here:


